# Using Social Network Analysis to Evaluate Health-Related Adaptation Decision-Making in Cambodia

**DOI:** 10.3390/ijerph110201605

**Published:** 2014-01-30

**Authors:** Kathryn J. Bowen, Damon Alexander, Fiona Miller, Va Dany

**Affiliations:** 1 National Centre for Epidemiology and Population Health, Australian National University, Canberra, ACT 0200, Australia; 2 Department of Resource Management and Geography, University of Melbourne, Carlton, VIC 3010, Australia; E-Mail: dta@unimelb.edu.au; 3 School of Social and Political Sciences, University of Melbourne, Carlton, VIC 3010, Australia; 4 Department of Environment and Geography, Macquarie University, Sydney, NSW 2109, Australia; E-Mail: Fiona.miller@mq.edu.au; 5 Department of Environmental Sciences, Royal University of Phnom Penh, Russian Confederation Blv., Phnom Penh, Cambodia; E-Mail: danyenvironment@gmail.com; 6 Institute of Sustainable Development and Architecture, Bond University, Gold Coast, QLD 4227, Australia

**Keywords:** governance, climate change adaptation, global health, Cambodia, social network analysis

## Abstract

Climate change adaptation in the health sector requires decisions across sectors, levels of government, and organisations. The networks that link these different institutions, and the relationships among people within these networks, are therefore critical influences on the nature of adaptive responses to climate change in the health sector. This study uses social network research to identify key organisational players engaged in developing health-related adaptation activities in Cambodia. It finds that strong partnerships are reported as developing across sectors and different types of organisations in relation to the health risks from climate change. Government ministries are influential organisations, whereas donors, development banks and non-government organisations do not appear to be as influential in the development of adaptation policy in the health sector. Finally, the study highlights the importance of informal partnerships (or ‘shadow networks’) in the context of climate change adaptation policy and activities. The health governance ‘map’ in relation to health and climate change adaptation that is developed in this paper is a novel way of identifying organisations that are perceived as key agents in the decision-making process, and it holds substantial benefits for both understanding and intervening in a broad range of climate change-related policy problems where collaboration is paramount for successful outcomes.

## 1. Introduction

Partnerships across multiple sectors and types of organisations that link organizations and individuals are considered crucial for effective climate change adaptation [1,2]. However, research, policy and advocacy communities have little understanding of how different organisations work together and combine their power to influence policy decisions and implementation, specifically in relation to health and climate change adaptation. An understanding of this broad governance structure is necessary given that the effects of climate change on health will arise predominantly via impacts on sectors not directly linked to health, including agriculture, water, transport and disaster risk reduction. This complicated web of decision-making can often result in a neglect of health concerns and confirms the need for involvement of multiple sectors and scales for decision-making. Also important here is acknowledging the increasing presence of actors ‘beyond the state’ such as non-government and private organisations, who are becoming more active in climate change adaptation. 

Consideration of the articulation of equity, influence and power should illuminate how decisions are made and their policy context [3]. Importantly, if an understanding of decision-making processes is possible, this can allow the identification of leverage points in order to assist advocacy efforts to support more equitable approaches to adaptation policy. Of interest here from a public health perspective is the potential to ultimately influence policy that will improve health equity. This strategic focus of identifying access points may have broader benefits than responding to the health effects of climate change; indeed, if we are to address the health effects of climate change, we ultimately need to address the underlying health inequities that climate change will exacerbate [4,5]. The current and continuing influx of adaptation funding is one opportunity to intensify activity to improve global health, and the health community needs to respond to this new funding with a view to addressing current and climate-sensitive burdens of disease [6].

An incorporation of the public health profile of a population is crucial when trying to understand and improve climate change adaptation. Likewise, an incorporation of the public health sector (including Ministries of Health, health-related non-government organisations and other health-related agencies) is important for inclusive adaptation decision-making. This consideration of the health sector necessitates the development of cross-sectoral adaptation activities to address direct and indirect health risks arising from climate-related events. For example, the direct health effects that arise from an extreme event such as a storm surge include an increase in water-borne disease if coastal sanitation systems are overwhelmed, while indirect effects can include a loss of agricultural productivity and a consequential increased rate of malnutrition. It is vital here that the rural development, agriculture and water sectors are all actively involved in formulating cross-sectoral adaptation activities to reduce the risks to such climate threats to health.

Although a multi-sectoral governance approach is necessary for effective and efficient climate change adaptation, this is not generally the normal operating approach of governments or, indeed, of many non-government organizations. This is despite the recognition that vulnerability to the health effects of climate change can be reduced by strengthening governance efforts [7] and by understanding governance structures (including decision-making processes), the pathways that lead to policy development and implementation within and between different sectors can be clarified [3,8,9].

This paper aims to describe the actual social networks underpinning climate change adaptation decision-making relevant to the health sector, as reported by stakeholders themselves. The findings are structured around three main factors that may enable (or inhibit) cross-sectoral and cross-organisational collaboration for climate change adaptation relevant to health: the extent of contact between state and non-state actors; the significance of informal networks; and the role of bridging organisations. A fourth factor, social capital, is not explicitly measured in this study but can be extrapolated from the social network research findings as a whole. For an expanded background discussion of these factors, see Bowen *et al.* [10].

The context for this study is Cambodia in Southeast Asia. Cambodia is a least-developed country that already confronts considerable challenges in the area of health [11] particularly in relation to child and maternal mortality, malnutrition and water-borne diseases. Cambodia is also considered highly vulnerable to the impacts of climate change [12,13,14].

In recognition of the above the Cambodian government has put in place a broad policy framework for addressing climate change adaptation. This is anchored in the Climate Change Department in the Ministry of Environment. The policy framework for climate change adaptation is based upon the National Adaptation Program of Action (NAPA), which will be superseded in the near future by the Second National Communication. The following section briefly provides an overview of the main factors that may enable (or inhibit) cross-sectoral and cross-organisational collaboration for climate change adaptation relevant to health.

### 1.1. Social Networks and Social Capital

Social capital is vital for adaptive governance [15] and is integrally linked to both the health of the natural environment and the human population. Social capital is broadly understood here as the social bonds and norms that contribute to social cohesion [16]. Securing livelihoods and maintaining wellbeing (at least partly) results from levels of social capital that enhance shared access to resources [17]. Further, it has been argued that community-based adaptation has social capital at its core [18].

The links between social capital, health (in particular mental health) and climate change have begun to be explored (see review in [19]), but the links between these factors and governance have not yet been thoroughly examined. The evaluation of social networks can serve as a method to explore and assess elements of social capital, given the importance of understanding the role of social networks in enhancing communities’ adaptive responses to environmental change and in supporting governance mechanisms [20,21]. Four central aspects of social capital are: relations of trust; reciprocity and exchanges; shared rules, norms and sanctions; and connectedness, networks and groups [22].

### 1.2. Actors beyond the State

The past decade has seen greater recognition of actors (both individuals and organisations) that exist beyond the traditional decision making structures and processes that occur within a nation state. Recognition of these ‘actors beyond the state’ underlines the importance of understanding social networks in a more holistic and systems-based approach as we come to accept the increasing relevance of non-state actors in influencing environmental governance processes [23] and more general governance processes [24]. Such actors include donor countries, international non-government organisations, development banks and the United Nations; all of whom are increasingly focusing attention on enhancing financial and technical support for climate change adaptation initiatives. While this may seem a positive step in terms of the increase in financial commitments, the influx of non-state actors involved in climate change adaptation presents challenges for the development of adaptation strategies that are complementary, avoid duplication of time, money and effort, and understand the complexity of the recipient country setting that is selected for their funding.

### 1.3. Informal Networks

Informal networks or ‘shadow networks’ are as essential as formal networks to consider when evaluating governance structures and decision-making processes [25], as they are important for the development of new ideas and creativity, and for the flow of information [2]. However, shadow networks have been the focus of research mainly in the field of social-ecological system governance to date, which has not explicitly included the health sector. Although informal networks may present layers of partnerships that do not conform to the more structured formal decision-making processes, it may be that the unstructured nature of these relations is as important for long-term capacity to adapt to global environmental change as much as the formal organisational structures [25]. This may be because informal networks reflect social relations more strongly, rather than political or bureaucratic relations that can be subject to greater political influence and manipulation. Another reason for the importance of informal networks is their potential to respond more quickly to changes in the political and social environment due to their unstructured nature, which is generally not reliant on a centralised level of control.

### 1.4. Bridging Organizations

It may not always be that the organisations sitting within the centre of decision-making processes yield all the influence. Bridging organizations also have an important role to play, as they link groups, networks and organizations across levels and create the right links between individuals, issues and timing [15,26]. The emergence of bridging organizations seems to lower the costs of collaboration by accessing and consolidating various avenues of knowledge and interest to respond to social-ecological change [15]. Although bridging organisations have primarily been the focus of research within the field of socio-ecological change, an understanding of bridging organizations is also useful for the health arena—perhaps even more so, given that the health effects are strongly determined by actions in sectors other than the health sector. Bridging organizations play an important role in climate change adaptation because although policy development and funding is generally conducted at a central government level, adaptation activities occur on a local scale, often driven by local governments [1,27,28,29] with other local organisations also involved. Organizations that act as links between these different scales may therefore increase the likelihood of inclusive and effective decision-making processes for adaptation policy and activity; ultimately such decision-making will contribute to more sustainable adaptation outcomes.

In this study, social network research was employed to understand these four components of governance—social networks and capital, actors beyond the state, informal networks and bridging networks—in the case of developing health-related climate change adaptation strategies. Social network research allowed us to map the organisations identified as active in the field as well as to understand the linkages between different decision-making relationships both within and between relevant ministries, NGO, research institutions and private organizations, across a wide variety of sectors.

Researchers in the field of social epidemiology have a strong history of using social network analysis to understand the spread of health conditions through social networks. Such studies have investigated a variety of conditions through social networks, such as the use of alcohol and other drugs [30,31,32], obesity [33] and back pain [34] to happiness [35] and social capital [36]. However, the use of social network research as a tool to understand the decision-making processes involved in health-related policy development is much less developed, and is the focus of this study.

## 2. Method

This study is one element within a larger research project exploring health and water vulnerability assessments and adaptation activities in the Asia Pacific region. Ethics approval for the study was obtained from the Australian National University’s Human Research Ethics Committee.

A total of 44 in-depth semi-structured interviews with key stakeholders were conducted between April and August 2009. The majority (41) were conducted in the capital of Cambodia, Phnom Penh, and a small number (3) were conducted in Ratanakiri, a province in northeast Cambodia. The stakeholders were purposively sampled, using a combination of expert-sampling and snowball approaches. Stakeholders represented a broad range of sectors, as well as types of organisations. Interviews were conducted by members of the research team using an interview guide, digitally recorded, transcribed and (where necessary) translated into English for analysis.

The objective of social network research and analysis in this study was to assess decision-making pathways and processes in relation to the development of adaptation options, and describe and measure the bonds between actors and sectors. We began by using a name generator to collect data on which organisations agencies formally partner with on health and climate related issues and by asking respondents to indicate how important they regarded these partnerships for their work in climate change. A second name generator was then used to collect data on informal partnerships, while a third was used to identify which agencies respondents considered most influential in shaping decision-making on adaptation strategies concerning the health effects of climate change in Cambodia. The specific questions asked of the respondents that is reported here were: (i) which agencies do you currently partner with on health and climate change related issues?; (ii) list the top ranking organizations with the most influence (“influence” is defined as a demonstrated capacity to do one or more of the following: shape ideas about policy, initiate policy proposals, substantially change or veto others’ proposals, or substantially affect the implementation of policy in relation to health. Influential people are those who make a significant difference at one or more stages of the policy [37]) in decision-making on adaptation strategies concerning the health effects of climate change in Cambodia; and (iii) which agencies do you currently partner with on an informal basis on climate change adaptation? A single count (or ‘tie’) was assigned to each organization that was identified.

## 3. Results and Discussion

Results are presented here firstly describing the formal networks, followed by the informal networks. The total number of respondents for the social network research was 30 (from an initial sample of 44) (Table 1). This was stratified by sector (health, water, agriculture, disaster, development, other sectoral ministries, other) and type of organisation (government, non-government) in order to explore differences between these sub-groups.

**Table 1:** Stakeholders who responded to social network questions stratified by sector and organization.

Sector Type of organisation	Health	Water	Agriculture	Disaster	Development ^a^	Other sectoral ministries ^b^	Other ^c^	Sub-total
**Government**	2	3	1	1	0	4	0	**11**
**Non government organisation**	2	1	2	3	10	0	1	**19**
**Sub-total**	**4**	**4**	**3**	**4**	**10**	4	1	
***Total***	***30***							

^a^ *Development* indicates organisations that work across more than one sector. This group was comprised of bi/multilateral development partners and NGOs. ^b^ *Other sectoral ministries* indicates Cambodian government ministries that sat beyond the stratification, and included ministries such as the Ministry of Education and Youth, Ministry of Environment, Ministry of Economics and Finance. ^c^ *Other* indicates organisations that sat beyond the stratification.

**Figure 1:**
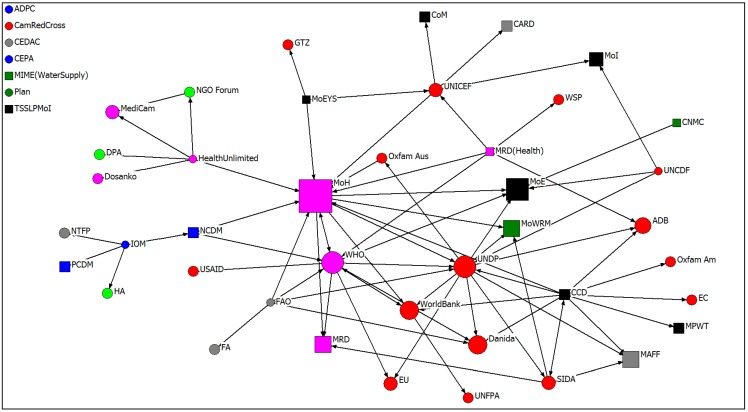
Health and climate change partnerships in Cambodia.

Figure 1 maps the formal health and climate-change related partnerships identified by the 30 organisations we interviewed in Cambodia. When more than one individual was interviewed from the same organisation, we chose the individual most relevant (and most senior if necessary) in order to maintain an equal representation of organisations. Each tie represents a partnership while each node represents an agency. Government actors are depicted by square nodes while non-government actors are represented by circles. The size of the nodes represents the number of ties directed towards each agency with the larger, more prominent organisations automatically grouped towards the middle of the graph. Node colour represents the sector each agency is located within, with the coding as follows: Agriculture (grey); Development (red); Disaster (blue); Health (pink); Water (dark green); Other Ministry (black); and Other (light green). The nodes in the upper left hand corner represent agencies that were interviewed who did not identify any partners. Overall, 27 of the 30 organisations we interviewed indicated that they were involved in at least one formal partnership related to health and climate change issues, with the mean being 2.18. Seventy-three partnerships were identified involving 48 organisations, the latter including government departments, UN agencies, environmental groups, and agriculture.

A simple visual examination of Figure 1 reveals at least three quite prominent characteristics. Firstly, we can see that a large number of the partnerships identified involve a relatively small number of key organisations. These include the Ministry of Health (MoH), the Ministry of Environment (MoE), the World Health Organisation (WHO), the United Nations Development Programme (UNDP) and the Danish International Development Agency (DANIDA). These groups are not only centrally placed within the network, but are also quite densely integrated themselves. Interestingly though, this core group of organisations appears to be surrounded by an outer circle of bridging agencies which act as a link between more peripheral organisations and the main group. The bridging agencies include UNICEF, the Ministry of Rural Development (Health Unit), the Climate Change Department (CCD), advocacy group Health Unlimited, and the UN agency Food and Agriculture (FAO). Finally, we can see quite a lot of boundary-spanning behaviour in the network, with many partnerships linking agencies from different sectors and a large number linking different types of organisation.

### 3.1. Centrality and Network Prominence

Mapping and ‘eyeballing’ social relationships in this manner provides a useful starting point for analysis of the structure and composition of the health and climate change partnerships network and the relative power and position of individual actors within it. Social network analysis though provides us with a range of specialised measures which enable us to examine these kinds of characteristics in a more sophisticated and nuanced manner.

Being more central in social networks potentially conveys all manner of social benefits including increased access to resources such as information and greater influence or control over how these resources are distributed [38,39,40]. In this context, social network analysis provides a relatively simple tool—‘in-degree centrality’—which we can use to measure how central or prominent individual actors are within a given network [41]. In a directed graph such as the health and climate change partnership network, the raw in-degree score simply measures the number of ties directed towards each organisation in the network from other organisations. The underlying assumption being that those more prominent in the network occupy positions which potentially convey strategic advantage and influence.

Table 2 lists the ten most prominent organisations identified in the health and climate change partnership network, with the in-degree score indicating the total number of partnerships with each organisation nominated by those interviewed (a normalized score which is calculated by dividing the number of ties received by the maximum possible number is also provided to enable comparison across different sized networks). As the data clearly indicates, the Ministry of Health is by far the most prominent partner in the network, with just over one in five of the organisations interviewed involved in a formal partnership arrangement with this department. The Ministry of Environment was also heavily nominated as were the UNDP and the World Health Organisation. The most notable absence in the top ten most prominent partners was that lack of non-governmental organisations.

**Table 2:** Health and climate change partnerships in Cambodia: Top10 most prominent partners.

	Sector	InDegree	Norm_Indeg
MoH	Health	10	21.28
MoE	Other Sectoral Ministries	5	10.64
UNDP	Development	5	10.64
WHO	Health	5	10.64
Danida	Development	4	8.51
WorldBank	Development	4	8.51
ADB	Development	3	6.38
MAFF	Agriculture	3	6.38
MoWRM	Water	3	6.38
MRD	Health	3	6.38

Half of the most prominent health and climate change partners were Cambodian ministries (MoH, MoE, MAFF, MoWRM and MRD). Four of the ten most prominent agencies were from the Development sector with a further three from the Health sector. The prominence of these two sectors is reflected in the data provided in Table 3 which provides a breakdown of the percentage of ties directed towards agencies and organisations involved in climate change and health related partnerships according to the sector in which they are located. As the table shows, almost one-third of partnership ties (32.88%) nominated by respondents were directed towards health-related organisations, with a slightly lower proportion (31.50%) directed towards the development sector, such as UNDP, the World Bank and DANIDA. Most other sectors were relatively minor players.

**Table 3:** Climate change and health-related partnership ties by sector.

Sector	Ties (raw)	(%)
Health	24	32.88
Development	23	31.50
Other sectoral ministries	14	19.18
Disasters	6	8.22
Agriculture	5	6.85
Other	1	1.37
Water	0	0
Total	73	100.00

### 3.2. Cross-Sectoral Networking

Cross-sectoral networking is crucial for an area such as climate change and health, as the health effects of climate change will predominantly arise via other sectors, such as water and agriculture. To measure the extent of cross-sectoral networking present in the health and climate change adaptation field we calculated the percentage of partnership ties directed across sectoral boundaries for the network overall; as well as a breakdown of this figure by agency sector; and by state/non-state sectors (Table 4). Looking firstly at the network as a whole, we can see that a larger majority of partnerships in the health and climate change adaptation field are cross-sectoral (72.60%), with just over a quarter involving agencies from the same sector as the respondent. More than 85 per cent of partnerships nominated by respondents from ‘Other Sectoral Ministries’ and 80 per cent of partnerships nominated by respondents from ‘Agriculture’ were to other sectors. Even ‘Health’ which was the least cross-organisational sector in terms of its partnership profile recorded over 70 per cent of external ties, suggesting that the climate change adaptation network is quite heterogeneous.

This boundary-spanning networking behaviour extends to partnership formation across the government/non-government divide. More than half of all partnerships were between state and non-state organisations, and more than one-third of all partnerships were between non-state organisations. In contrast, less than 14 per cent of partnerships were between state actors.

Table 5 lists the ten organisations whose climate change and health related partnership profiles are most externally focused ranked by the percentage of partnership ties directed towards organisations located in other sectors. The data shows that the Climate Change Department is among eight organisations whose formal partnerships are all based around ties to agencies in external sectors.

**Table 4:** Ties between different sectors and types of organizations.

**Type of partnership (sector)**	**Number of ties**	**Number of ties (%)**
Cross-sectoral	53(73)	72.60
Sector specific	20(73)	27.40
Health	17(24)	70.83
Development	14(23)	60.87
Other Sectoral Ministries	12(14)	85.71
Disasters	4(6)	66.67
Agriculture	4(5)	80.00
Other	1(1)	100.00
Water	-	-
**Type of partnership (organisation)**		
State – non-state	38	52.1%
Non-state – non-state	25	34.2%
State – state	10	13.7%

**Table 5:** External partnerships: Top 10 agencies (% of partnership ties).

Name	Sector	Ties (raw)	External (%)
CCD	Other Sectoral Ministries	10	100
CNMC	Water	1	100
MoEYS	Other Sectoral Ministries	3	100
NCDM	Disasters	2	100
NGO Forum	Other	1	100
Oxfam Aus	Development	1	100
Sida	Development	4	100
UNICEF	Development	4	100
FAO	Agriculture	4	80
WHO	Health	6	75

### 3.3. Organisational Influence

Understanding the various levels of influence that organisations are perceived to hold is important in order to gauge where best to target advocacy efforts; in this case for strengthening action on health and climate change adaptation activities and policies. Stakeholders were asked to nominate the organisations with the most influence in decision-making on adaptation strategies concerning the health effects of climate change in Cambodia. The 30 respondents made 93 nominations overall, with 20 influential organisations identified. Of these, just 14 received multiple nominations. Table 6 lists these organisations along with the raw number of nominations and a normalised figure which shows the percentage of total nominations possible received by each organisation. As the results shows, influence is highly centralised within the hands of government, and to a lesser extent, the World Health Organisation, with the remainder of the organisations displaying only a small level of influence. The Ministry of Health was clearly identified as the most influential organisation in relation to the development of health-related adaptation strategies, with 72% of all respondents nominating it as influential. The Ministry of Environment was ranked second with 18 nominations out of a possible 36 with the WHO ranked third with 33.33 per cent. There is a large gap in terms of perceptions of influence between these first three organisations and the remainder.

**Table 6:** Influence nominations for health and climate change organisational influence in Cambodia.

Organisation	Influence nominations	Normalised Influence Nominations#
MoH	26	72.22
MoE	18	50.00
WHO	12	33.33
NCDM	5	13.89
MAFF	4	11.11
MoWRM	4	11.11
CoM	3	8.33
UNDP	3	8.33
MoEF	2	5.56
MoI		
MRD		
NCCC		
NGOForum		
WorldBank		

# = Total nominations/N-1 × 100.

**Table 7:** Influence nominations for health and climate change organisations: Sector.

Sector	Influence Nominations (raw)	Total (%)
Development	31	33.33
Disasters	18	19.36
Health	12	12.90
Other sectoral ministries	12	12.90
Agriculture	10	10.75
Water	7	7.53
Other	3	3.23

Table 7 provides a breakdown of the influence nominations by sector. As the data shows, one-third of all nominations (33.33 per cent) were for organisations within the ‘Development’ sector, with a further 19.35 per cent directed towards those in ‘Disasters’. The health sector captured only 12.90% of the sectoral-based influence nominations. Beyond these two sectors, perceptions of influence were relatively widely dispersed across the remaining eight sectors.

### 3.4. Influence and Centrality

To explore the relationship between involvement in partnerships and influence, we compared the in-degree centrality scores of each organisation nominated in the health and climate adaptation partnerships network with the influence nominations recorded (Table 8). As previously noted, according to social network theory, we would expect to see a close positive relationship between higher levels of prominence, influence and/or power and in-degree centrality within the partnership networks [41,42]. The data clearly bears this relationship out, albeit we are measuring perceptions of influence.

**Table 8:** Partnership centrality and influence: Top 10.

Partnership Centrality		Influence	
Organisation	Norm_Indeg	Organisation	Nomination (%)
MoH	21.28	MoH	72.22
MoE	10.64	MoE	50.00
UNDP	10.64	WHO	33.33
WHO	10.64	NCDM	13.89
Danida	8.51	MAFF	11.11
WorldBank	8.51	MoWRM	11.11
ADB	6.38	CoM	8.33
MAFF	6.38	UNDP	8.33
MoWRM	6.38	MoEF	5.56
MRD	6.38		
		MoI	
		MRD	
		NCCC	
		NGOForum	
		WorldBank	

Of the ten most central organisations in the partnership network eight were also amongst the top ten ranked actors in terms of influence. The Ministries of Health and Environment were ranked first and second respectively on both centrality and influence measures, with the WHO also ranked in the top four on both measures. Danida and ADB are the only two top 10 ranked organisations in terms of partnership centrality that were not highly ranked in terms of influence. On the other side of the equation there were four organisations who were ranked top 10 in terms of perceived influence who were not prominent in climate change and health partnerships. Of these, NCDM was the most highly ranked with 13.89 per cent of all influence nominations made, with the CoM also seen as relatively influential. Both of these organisations received just the single partnership nomination. This very close relationship between the partnership centrality of organisations and their perceived influence in the eyes of the stakeholders interviewed is confirmed by simple correlation analysis which shows a Pearson Correlation Coefficient of 0.82 significant at *p <* 0.001.

### 3.5. Informal Partnerships

**Figure 2:**
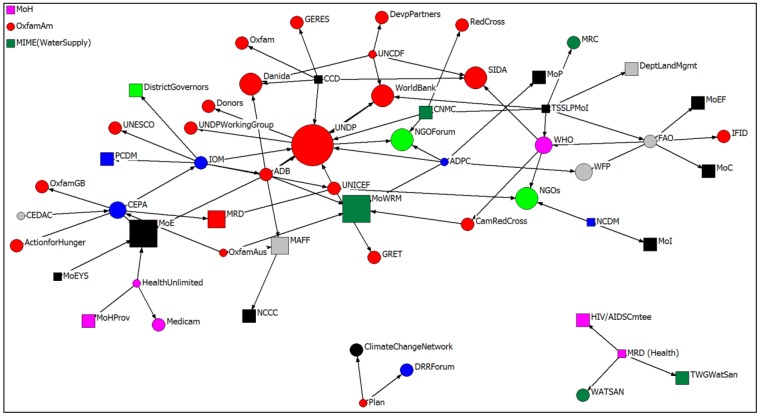
Informal partnerships in the field of climate change adaptation in Cambodia.

The evaluation of informal partnerships aims to identify the existence of shadow networks – that is, those networks that may not appear in a formal representation in the previous figures, but play an important role in influencing decisions in more informal ways. Stakeholders were asked to nominate the informal partnerships they had with other organisations working in climate change adaptation (not necessarily health-specific). Out of the 30 interviews conducted, 59 organisations (nodes) were identified, and 74 ties were nominated (Figure 2). A visual examination of Figure 2 reveals at least two clear characteristics. Firstly, there are a greater number of organisations identified than in the formal networks, and secondly, similarly to the formal networks, there is a high degree of partnerships that cross sectors and organisational types. The majority of stakeholders identified the important role of informal partnerships. The UNDP was identified as the organisation with the most informal ties, followed by the MoE and MoWRM (Table 9). There was a tight clustering of organisations that completed the top organisations with the most informal ties, which included only four Cambodian ministries.

**Table 9:** Informal partnerships with organisations working in climate change adaptation: Most prominent/central partners.

	Sector	InDegree	Norm_Indeg
UNDP	Development	8	6.9
MoE	Other sector ministries	5	4.31
MoWRM	Water	4	3.45
Danida	Development	3	2.59
NGOForum	Other	3	2.59
Other NGOs	Other	3	2.59
Sida	Development	3	2.59
WorldBank	Development	3	2.59
CEPA	Disasters	2	1.72
MAFF	Agriculture		
MRD	Other sector ministries		
WFP	Agriculture		
WHO	Health		

Examples of ways that people engaged included informal meetings, such as the monthly meetings of the climate change group, attended by 30 organisations including government agencies, development banks, UN agencies and local NGOs. The Disaster Risk Reduction Forum which meets regularly as well as the information forum that was facilitated by the UNDP were also nominated as important occasions for informal networks. Friends were also indicated by a number of stakeholders as an important informal network (e.g., at MoWRM—provide meteorological information in a timely manner).

Table 10 provides a breakdown of the percentage of ties directed towards agencies and organisations involved informally in climate change adaptation partnerships according to the sector in which they are located. As the table shows, almost one-third of partnership ties (32.43%) nominated by respondents were directed towards development-related organisations, with more than one-quarter (25.68%) directed towards the disaster sector. The health, agriculture and water sectors do not seem to play an important role in informal adaptation partnerships.

**Table 10:** Climate change adaptation informal partnership ties.

Sector	Ties (raw)	(%)
Development	24	32.43
Disasters	19	25.68
Other sector ministries	12	16.22
Health	9	12.16
Agriculture	7	9.46
Water	3	4.05
Other	0	0
Total	74	100

### 3.6. Cross-Sectoral Networking

In the same way that we measured the extent of cross-sectoral networking present in the formal health and climate change networks, we also calculated the percentage of informal partnership ties directed across sectoral boundaries for the informal climate change adaptation network overall; as well as a breakdown of this figure by agency sector; and by state/non-state sectors (Table 11). In terms of the network as a whole, a strong majority of informal partnerships in the health and climate adaptation field are cross-sectoral (72.97%). The remainder (27.03%), partner informally only within their own sector. All sectors except for development displayed a large majority of partnerships that lay beyond their own sector.

In terms of partnerships across state and non-state actors, the highest proportion of informal partnerships were between non-state actors (50%), with partnerships between state and non-state actors showing a fairly high representation of 39.19%. State to state partnerships were low (10.81%).

Clear differences are evident between the formal and informal partnership social network maps. The most prominent difference is that the Ministry of Health (central level) was not identified in any informal partnerships, despite being identified as the organisation with the most nominations of formal partnerships. The UNDP maintained its high level of partnership activity in both formal and informal networks. In both formal and informal social networks, the majority of the organisations identified in the top ten were UN or bi/multilateral agencies (six of the top ten in each network). Only one health-specific organisation, the WHO, was identified in the top ten in the informal network.

**Table 11:** Informal ties between different sectors and types of organizations.

**Type of partnership (sector)**	**Number of ties (74)**	**Number of ties (%)**
Cross-sectoral	54(74)	72.97
Sector specific	20(74)	27.03
Agriculture	6(7)	85.71
Development	10(24)	41.67
Other Sectoral Ministries	11(12)	91.67
Disasters	17(19)	89.47
Health	6(9)	66.67
Water	3(3)	100
Other	-	-
**Type of partnership (organisation)**		
State–non-state	29	39.19%
Non-state–non-state	37	50.00%
State–state	8	10.81%

No differences were present in terms of the extent of cross-sectoral networking between the formal and informal networks. Partnerships were stronger between non-state actors in the informal networks, but partnerships between state and non-state were still quite high in both formal and informal networks.

### 3.7. Discussion

The use of social network analysis in this study provided an opportunity to elucidate decision-making processes around the development of health-related climate change adaptation policy in Cambodia. In particular, the study identified organisations that were perceived to be central to the decision-making processes in both formal and informal networks. 

In terms of partnerships within the field of health and climate change in Cambodia, the finding that there were multiple links between different sectors and types of organisations is promising for a cross-cutting issue such as health and climate change. The (small number of) organisations with the most partnerships were not confined to a particular sector or type of organisation, rather, they were a combination of Cambodian Ministries, and UN and bilateral agencies. In addition, half of the top ten most prominent organisations in terms of partnerships were Cambodian ministries, which is an important finding, and points to the critical role of Cambodian organisations in decision-making processes despite the presence of many donors and high level of dependence on aid. 

Although the high level of the Ministry of Health’s centrality may appear logical, it is somewhat surprising, as the MoH does not place a high priority on climate change, nor was it represented strongly within the first NAPA. It also has not been central in climate change adaptation funding negotiations, e.g., with the UNDP or the World Bank’s Pilot Program for Climate Resilience and the process of identifying projects for funding through this initiative.

The presence of bridging organisations as key players that facilitate partnerships extending beyond the central active layer is important, as these can also be used as potential avenues for advocacy efforts.

In terms of types of partnerships, the majority of these were cross-sectoral, along with strong partnerships between government and non-government organisations. This indicates that there is a healthy level of cross-pollination occurring between not just different sectors, but also types of organizations—actors beyond the state—a finding which is vital for the sorts of collaborative activities required to adequately respond to health and climate change issues. This finding could also reflect that there is a well-functioning Technical Working Group on health in Cambodia that meets regularly to discuss priority health concerns.

The highly centralised nature of perceived influence that was identified as lying within Cambodian government ministries (particularly health and environment) indicates that despite the increasing funding on climate change adaptation (as well as Cambodia’s heavy reliance on ODA), these factors do not appear to have diluted the influence that local decision-making bodies have on climate change adaptation policy. However, this finding is not in accordance with other research (e.g., [43], which has found non-government organisations (including donors, UN agencies, traditional NGOs) do bear a considerable amount of influence in policy development. Further research is needed that can illuminate in more detail the complicated nature of the donor/government relationship. In addition, this type of interview response is challenging to collect, given its reliance on minimising interviewing biases, particularly response bias. In the context of Cambodia, it may be that this question eliciting influence was difficult for stakeholders to assess, or an alternative explanation is that cultural factors (such as respecting the authority of the government and official hierarchies) were seen as more important to conform to rather than responding in a transparent manner to the interview question. 

In order to surmount this issue, a dissemination workshop was held to discuss the findings, with opportunities for individual and group reflection on the results. Stakeholders who attended did not express any objections to the social network findings, with some explaining that the prominence of government in relation to influencing policy development was accurate, and corresponded with the explicit mandates of some development partners (such as the World Bank) who seek to support, rather than dictate, internal domestic policy in Cambodia.

Despite the potentially ambiguous findings in terms of organisational influence, the finding that stakeholders considered the MoE as able to advocate for health and adaptation, even though that is not regarded as its ‘core business’ was important. This is important as it is within the MoE that the main climate change policies and activities emerge. The finding that there was a close correlation between organisations that had many partnerships and those that were perceived to be influential points to the symmetry in these two networks; neither influence or partnership activity can be seen as mutually exclusive. 

The Ministry of Health’s lack of presence within the informal networks, despite its prominence and influence in the formal networks, suggests that ‘health’ was not highly prioritized within the climate change adaptation sphere, given that the informal climate change adaptation networks represent informal adaptation institutions. Interpreting this further, perhaps the informal networks do not play as strong a role as they may in other situations, given that the MoH still scored the highest influence measure.

Although social capital was not explicitly measured in this study, the strong level of boundary-spanning networking behavior, as well as the presence of bridging organisations, suggest a high general level of social capital. Further research is needed to more specifically evaluate social capital.

## 4. Conclusions

The use of social network research in this study has highlighted the complicated and active nature of partnerships in relation to health and climate change adaptation in Cambodia. This research has shown that there is a very healthy level of partnerships reported in the field of health and climate change adaptation, although there are also areas to build on. Each of the elements outlined that effect collaboration—non-state actors, informal networks and bridging organizations—have shown to play an active role in the partnership and influence networks. The high level of involvement of Cambodian government organisations in these networks, coupled with involvement from development partners, including donors and traditional non-government organisations, sets the scene for further collaborative potential around developing health and climate change adaptation policy and activities that are necessarily cross-sectoral and multi-organisational. The implication of these findings for policy ownership is promising, as a high level of involvement of public institutions in the development of policies is crucial for the legitimacy of such policies. The extent to which civil society is engaged in this process, though not addressed in this research, is an additional element of importance to consider in this regard.

Barriers to progressing and supporting the cross-sectoral and multi-organisational partnerships within the field of health and climate change adaptation include: the highly fractured nature of international climate change adaptation funding; competition amongst different organisations for funding; and the slowness of the government in delivering its Second National Communication, which will outline the priorities for climate change adaptation. 

This technique reveals additional information to that of a traditional institutional analysis, as it allows a focus on the properties and characteristics of the network as a whole entity, providing a more integrated understanding than just an understanding of individual organizations. The value in this method is that it is a useful and engaging way to visually map and begin to understand the partnerships that exist in a fast-evolving field such as health and climate change. In addition, given the need for cross-sectoral and multi-organisational collaboration in climate change adaptation policy development, this approach provides a layer of analysis that can clearly visualise where networks exist, and where possibilities for intervention (such as advocacy activities) can occur. However, despite these positive elements of the social network analysis method, the method is not without limitations. This was particularly the case in relation to eliciting responses around the influence question that did not just conform to (i) a perceived ‘accurate’ response by the stakeholder (response bias) or (ii) cultural factors that discourage alternative views to the status quo (*i.e.*, the government necessarily commands the greatest level of influence). Approaches to minimise these limitations include a greater involvement and training of skilled researchers in collecting the data, a stronger emphasis on confidentiality and anonymity of responses, and testing alternative ways of devising the interview questions that incorporates a greater understanding of contextual and cultural issues. The existence of prior relations between researchers and participants, as was partly the case in this research, would also build the trust necessary to underpin the accuracy of such techniques.

This research is of significance considering the increase in adaptation financing to developing countries, especially least-developed countries—though actual funds fall well short of what is required and what has been pledged. Critically, the research can assist in identifying key organisations, and their relationships, that are perceived as key agents in the decision-making process. Greater understanding of these complex and dynamic relationships can improve our understanding of and intervening in a broad range of climate change-related policy problems where collaboration is paramount for successful outcomes.

## Acronym List

ADB: Asian Development Bank
ADPC: Asian Disaster Preparedness Centre
AusAID: Australian Agency for International Development
CARD: Council for Agriculture and Rural Development
CCD: Climate Change Department
CEDAC: Cambodian Centre for Study and Development in Agriculture
CEPA: Culture and Environment Preservation Organization
CoM: Council of Ministers
CNMC: Cambodian National Mekong Committee
Danida: Danish Development Agency
DPA: Development and Partnership in Action
DRRForum: Disaster Risk Reduction Forum
EC: European Commission
EU: European Union
FA: Forestry Administration of Cambodia
FAO: Food and Agriculture Organisation
GERES: Group for the Environment, Renewable Energy and Solidarity
GRET: Research Group on Technology Exchange
GTZ: German Development Agency
HA: Highlanders Association
IFID: International Financial Institutions Department
IOM: International Organisation for Migration
MAFF: Ministry of Agriculture, Forestry and Fisheries
MIME: Ministry of Industry, Mining and Energy
MoC: Ministry of Commerce
MoE: Ministry of Environment
MoEF: Ministry of Economics and Finance
MoEYS: Ministry of Education, Youth and Sport
MoH: Ministry of Health
MoI: Ministry of Interior
MoP: Ministry of Planning
MoPWT: Ministry of Public Works and Transport
MoWRM: Ministry of Water Resources Management
MRC: Mekong River Commission
MRD: Ministry of Rural Development
NCCC: National Committee for Climate Change
NCDM: National Committee for Disaster Management
NTFP: Non-Timber Forest Products Exchange Program
PCDM: Provincial Committee for Disaster Management
Sida: Swedish International Development Cooperation Agency
TSSLP: MoI Tonle Sap Sustainable Livelihoods Project, Ministry of Interior
TWGWatsan: Technical Working Group Water and Sanitation
UNCDF: United Nations Capital Development Fund
UNDP: United Nations Development Programme
UNFPA: United Nations Population Fund
UNESCO: United Nations Educational, Scientific and Cultural Organization
UNICEF: United Nations Children’s Fund
USAID: United States Development Agency
WATSAN: Water and Sanitation
WFP: World Food Program
WHO: World Health Organisation
WSP: Water and Sanitation Program
